# Tumefactive Demyelinating Lesion Mimicking Low-Grade Astrocytoma with a T2/FLAIR Mismatch Sign: A Case Report and Review of the Literature

**DOI:** 10.3390/diagnostics15243174

**Published:** 2025-12-12

**Authors:** Maria Karhu, Roberts Tumeļkāns, Dace Dzirkale, Kaspars Auslands, Can Özütemiz, Alīna Flintere Flinte, Arturs Balodis

**Affiliations:** 1 Faculty of Medicine, Riga Stradins University, 16 Dzirciema Street, LV-1007 Riga, Latvia; 2 Clinic of Neurology, Riga East University Hospital, LV-1079 Riga, Latvia; 3 Clinic of Neurosurgery, Riga East University Hospital, LV-1079 Riga, Latvia; kaspars.auslands@rsu.lv; 4 Department of Neurology and Neurosurgery, Riga Stradins University, LV-1007 Riga, Latvia; 5 Department of Radiology, University of Minnesota, Minneapolis, MN 55455, USA; 6 Department of Neurology, Pauls Stradins Clinical University Hospital, LV-1002 Riga, Latvia; 7 Institute of Diagnostic Radiology, Pauls Stradins Clinical University Hospital, LV-1002 Riga, Latvia; 8 Department of Radiology, Riga Stradins University, LV-1007 Riga, Latvia; 9 Faculty of Medicine and Life Sciences, University of Latvia, LV-1004 Riga, Latvia

**Keywords:** tumefactive demyelinating lesion, magnetic resonance imaging, low-grade astrocytoma

## Abstract

**Background and Clinical Significance:** Tumefactive demyelinating lesions (TDLs) are large demyelinating lesions that mimic intracranial tumors, posing a diagnostic challenge in both clinical presentation and conventional imaging. Distinguishing TDLs from central nervous system tumors can be challenging due to their similar imaging appearances. Specific magnetic resonance imaging (MRI) features such as open-ring contrast enhancement, mild mass effect, lack of cortical involvement, and rapid responsiveness to corticosteroids favor a demyelinating etiology of the lesion. This report presents a case of a tumefactive demyelination lesion showing a T2/fluid-attenuated inversion recovery (FLAIR) mismatch sign suggestive of a low-grade astrocytoma, focusing on imaging findings, therapeutic response, and diagnostic considerations. **Case Description:** A 63-year-old woman presented with headache, progressive speech impairment, and difficulty swallowing. MRI revealed a large lesion in the left frontal lobe with a T2/FLAIR mismatch sign, which initially suggested a low-grade astrocytoma. Additionally, the lesion was hypodense on noncontrast computed tomography (CT), did not show open-ring enhancement, and only had mild mass effect with perifocal edema. Given these conflicting imaging findings, a biopsy was considered; however, the patient declined the procedure and agreed to a follow-up. Corticosteroid therapy was initiated to reduce swelling, resulting in a significant reduction in the lesion within two weeks. A follow-up MRI confirmed near-complete regression of the lesion after two months. **Conclusions:** While a T2/FLAIR mismatch sign correlates with isocitrate dehydrogenase (IDH)-mutant 1p/19q non-codeleted astrocytoma, the dynamic radiological and clinical response to corticosteroids was more indicative of demyelination. This case highlights the importance of considering TDLs in the differential diagnosis of tumor-like brain lesions to avoid unnecessary invasive interventions like biopsy or surgical removal.

## 1. Introduction

Tumefactive demyelinating lesions (TDLs) are atypical variants of a demyelinating disease of the central nervous system that can resemble brain tumors both radiologically and clinically. These solitary or multifocal demyelinating lesions are larger than 2 cm in diameter, most commonly occurring in association with multiple sclerosis (MS), either during the course of the disease or more often as its initial manifestation [1,2].

Although TDLs may appear at any age, they are most often seen between the second and fourth decades of life with a female preponderance [1,3]. Onset at or after the age of 60 is distinctly uncommon but remains possible, which makes distinguishing TDLs from neoplasms even more challenging in this age group [1,4]. Among central nervous system (CNS) neoplasms, gliomas and primary CNS lymphomas (CNSLs) represent the most common differential diagnoses for tumefactive demyelinating lesions since they can share several clinical, histopathological, and radiological features [3,5].

Histopathologically, TDLs primarily exhibit extensive demyelination with relative axonal preservation, accompanied by lymphocytic inflammation, infiltration by foamy macrophages, and reactive astrocytosis containing multinucleated Creutzfeldt–Peters cells [6,7]. These features may mimic neoplasms due to hypercellularity, astrocytic pleomorphism, occasional necrosis, or cystic changes [1,7].

The clinical presentation varies and depends on lesion size and its location. TDLs often tend to localize in the supratentorial region, in the frontal and parietal lobes, and might cause motor, cognitive, sensory, and cerebellar symptoms [1,3]. Clinical presentation is nonspecific, and individual symptoms at presentation do not reliably distinguish TDLs from CNS neoplasms [5].

Brain MRI is the main modality for detecting TDLs, but it may not reliably differentiate TDLs from neoplasms, and biopsy might be required for a definitive diagnosis. On MRI, TDLs often show contrast enhancement in an open-ring pattern, none or mild mass effect, a variable degree of perifocal edema, and a lack of cortical involvement. The characteristic open-ring enhancement, in which the incomplete portion of the ring abuts the gray matter of the cortex or basal ganglia, is typical of TDLs. The enhancing component of the ring is regarded as the advancing front of demyelination and faces the white matter side of the lesion, while the non-enhancing central core represents a more chronic inflammatory process [8,9].

TDLs may also show varying patterns of contrast enhancement such as homogenous, heterogenous, patchy and diffuse, or cotton ball appearances [1]. Additionally, on diffusion-weighted imaging (DWI), a significant proportion of TDLs demonstrate peripheral restriction. Nevertheless, these radiological features are not specific and may overlap with findings seen in CNS neoplasms. Similarly, some tumors, such as primary CNSL, can resemble TDLs by having multiple small lesions with varying enhancement patterns [3,10,11].

A T2/FLAIR mismatch sign is generally reported as highly specific for isocitrate dehydrogenase (IDH) mutant 1p/19q non-codeleted astrocytomas, with specificity approaching 100% [12]. However, this specificity can be achieved only when the sign is assessed within the context of adult-type gliomas. Evaluation of this sign requires the presence of a hyperintense T2 signal and partial suppression on FLAIR except for a hyperintense peripheral rim at the lesion margins.

Several false-positive reports have been described in pilomyxoid astrocytoma, oligodendroglioma, H3K27M-mutant midline glioma, and low-grade astrocytoma harboring MYB rearrangement [13,14]. This radiological sign has also been reported in dysembryonic neuroepithelial tumors, suggesting that it should be interpreted with caution when encountered outside the context of low-grade astrocytomas [13]. The presence of the T2/FLAIR mismatch sign is exceptionally rare in TDLs and to the authors’ knowledge, only a few similar cases have been reported to date. Here we present a case most likely diagnosed as a tumefactive demyelinating lesion mimicking a low-grade astrocytoma, exhibiting a T2/FLAIR mismatch sign.

## 2. Case Report

A 63-year-old woman presented to the emergency department with a suspected cerebrovascular disorder. In the emergency department, the patient complained of headache, dizziness, and an inability to speak or write correctly. A neurological examination was performed; the patient was slow to respond. Meningeal signs were negative. Pupils were equal, reactive to light, and symmetrical. Cranial nerve exams were negative. Mixed sensory and motor aphasia and dysphagia were observed. No coordination disorders were seen. The patient reported that the headaches, dizziness, and a feeling of unsteadiness began around two weeks ago. A few days later, additional complaints emerged, including difficulty swallowing and speech disturbances, such as mixing up words and being unable to write words correctly. Past medical history was positive for hypothyroidism and osteoporosis.

**Figure 1:**
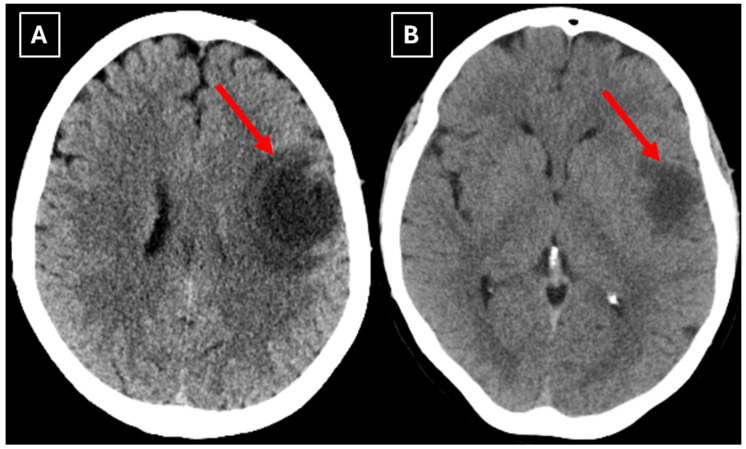
Native head CT without I/V contrast Images (**A**,**B**) show a. Hypodense lesion in the left hemisphere, basal parts of the frontal lobe. Measuring up to at least 3.5 cm in maximum size, with mild perifocal edema. Red arrow—indistinct formation.

A computed tomography (CT) scan of the brain revealed a 3.5 cm hypodense lesion in the left frontal lobe with mild perifocal edema (Figure 1). Due to the unclear nature of the lesion, additional examinations were performed to rule out oncopathology, including mammography and breast ultrasound, as well as chest and abdominal CT scans, all of which were negative for oncological findings.

**Figure 2:**
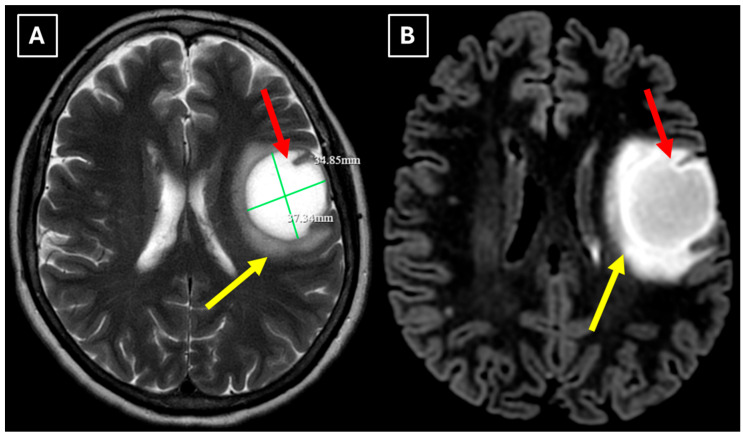
MRI scan of the brain. (**A**): Axial T2-weighted sequence shows a well-defined, homogenous, hyperintense mass-like lesion in the inferior part of the left frontal lobe, with involvement of the juxtacortical white matter and relative sparing of the cortical gray matter. The lesion measured 37.34 mm × 34.85 mm (AP × LL). Additionally, there is incomplete perifocal edema surrounding the lesion, extending to the white matter and away from the cortex. (**B**): Axial FLAIR sequence shows the lesion with a relatively more hypointense center and a hyperintense peripheral rim at the lesion margins. A T2/FLAIR mismatch sign was observed, making the likely diagnosis an infiltrative low-grade astrocytoma. All MRI images were acquired with the Philips “Ingenia Ambition X” 1.5T system. Red arrow—indistinct formation, yellow arrow—perifocal vasogenic edema.

**Figure 3:**
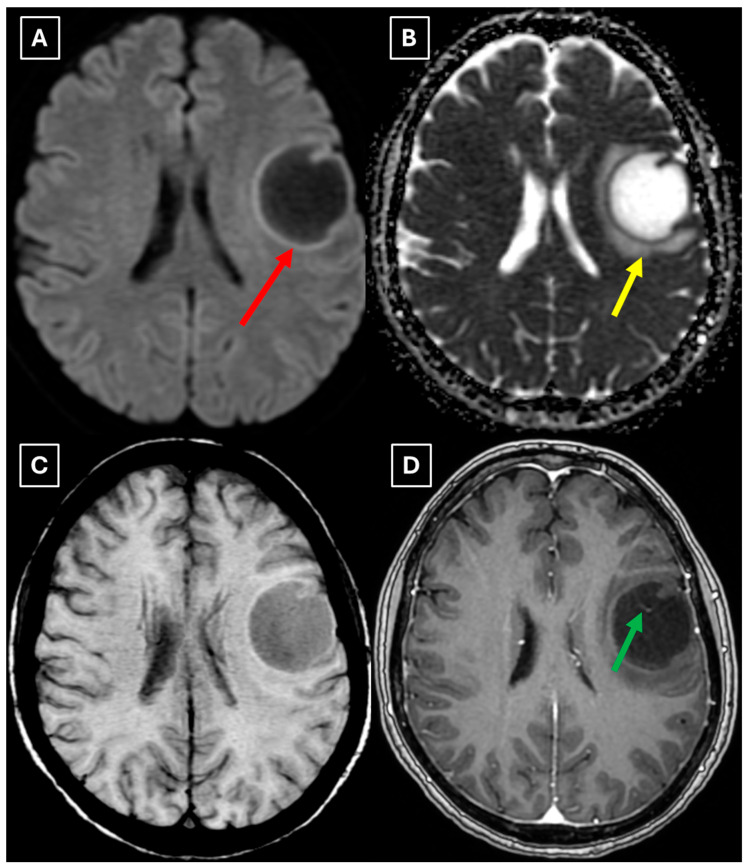
MRI scan of the brain. (**A**): Axial trace image of diffusion-weighted imaging (DWI) shows slight diffusion restriction in the lesion wall when correlated with (**B**): dark signal on apparent diffusion coefficient (ADC) maps in the wall of the lesion, with no central diffusion reduction observed. (**C**): In the corresponding axial slice of the susceptibility-weighted imaging (SWI) sequence, there is no evidence of blood products within the lesion. (**D**): On the axial postcontrast T1-weighted imaging, there is no contrast enhancement in the lesion, although a central vein-like structure is observed. Red arrow—diffusion restriction, yellow arrow—perifocal vasogenic edema, green arrow—central vein sign.

One week later, a brain MRI with intravenous contrast was performed, which revealed a well-defined subcortical lesion approximately 3.5 cm in diameter in the posterior basal parts of the left frontal lobe, accompanied by perifocal edema and sulcal effacement (Figure 2). No pathological contrast enhancement was observed (Figure 3). No other white matter lesion was identified to suggest signs of leukodystrophy, and no hemosiderin deposits were identified in the brain or meninges (Figure 3). Given the T2/FLAIR mismatch (Figure 2), the most likely diagnosis was infiltrative low-grade astrocytoma. The patient received mannitol anti-edema therapy, resulting in clinical improvement and a reduction in neurological symptoms. The case was discussed by a multidisciplinary team (neurologist, neurosurgeon, radiologist, oncologist, and pathologist). Surgical treatment and morphological verification of the tumor were recommended. A day later the patient was discharged from the hospital in a stable condition, with a recommendation to be admitted to the neurosurgery department for surgery in two weeks. She was prescribed dexamethasone tablets of 0.5 mg twice daily on an outpatient basis.

After treatment with glucocorticoids for two weeks, the patient was admitted for surgery. At admission, she had no complaints, and neurological status was practically normal: cranial nerves were within normal limits, there were no motor or sensory disturbances, muscle strength was 5/5 in all groups, and there were no coordination problems.

**Figure 4:**
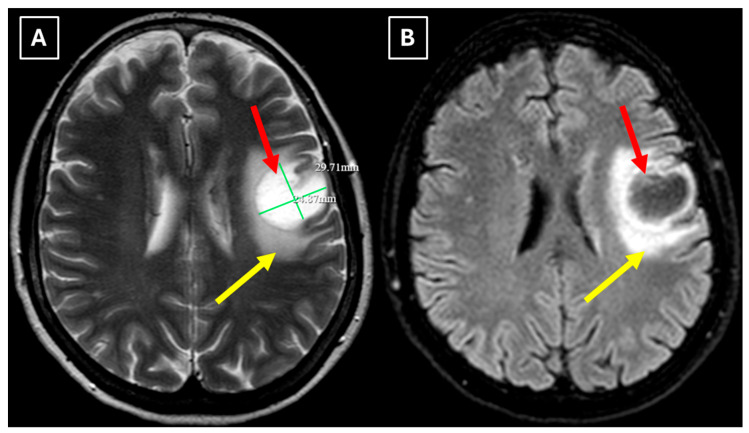
Two-week follow-up MRI scan of the brain. (**A**): Axial T2-weighted image shows a hyperintense and homogenous left frontal lobe lesion with well-defined borders. The lesion decreased from 37.34 mm × 34.85 mm (AP × LL) to 24.87 mm × 29.71 mm (AP × LL). A relatively extensive perifocal edema again persists, involving the white matter with relative spare of the white matter adjacent to gray matter; however, there is no mass effect or midline shift. (**B**): In the axial FLAIR the lesion appears with a more hypointense center and hyperintense peripheral rim at the lesion margins. Given the observed changes over a 2-week period and the decrease in the lesion’s size, an astrocytoma is considered less likely. Red arrow—indistinct formation, yellow arrow—perifocal vasogenic edema.

**Figure 5:**
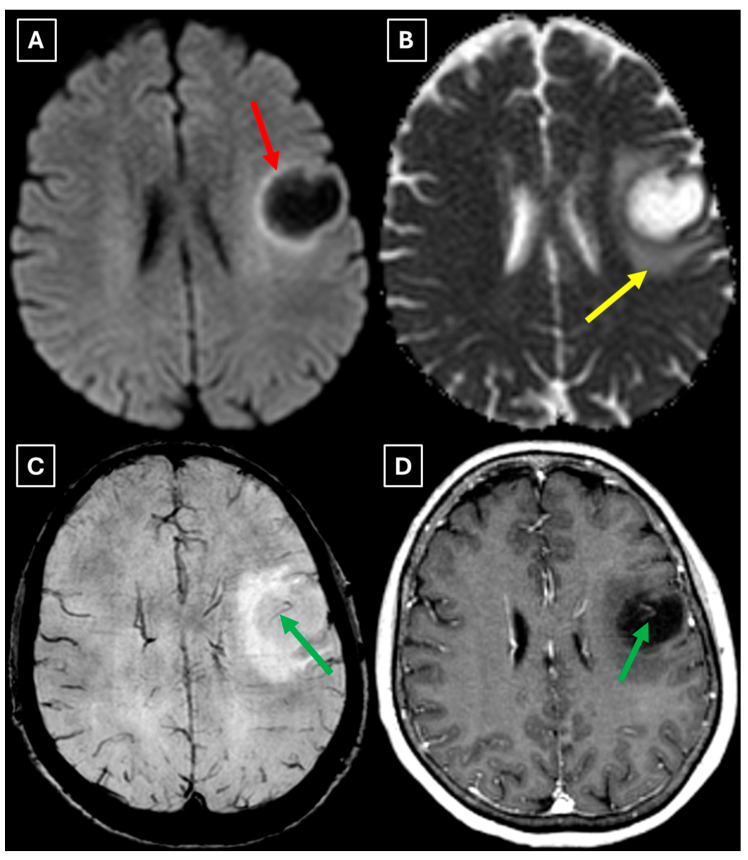
Two-week follow-up MRI axial scan of the brain. (**A**): Axial trace from diffusion-weighted imaging shows a higher signal along the peripheral parts of the lesion with the absence of cytotoxic edema when correlated with (**B**): the ADC map. (**C**): Central vein sign is positive on the susceptibility-weighted imaging sequence and (**D**): axial postcontrast T1-weighted sequence. These findings were not typical for low-grade astrocytomas. Red arrow—diffusion restriction, yellow arrow—perifocal vasogenic edema, green arrow—central vein sign.

A repeat MRI before surgery showed that the lesion had reduced in size with no pathological contrast uptake persistence, with surrounding perifocal edema (Figure 4 and Figure 5). Given the improved results, the medical team suspected a demyelinating disease rather than a tumor and surgery was canceled. For diagnostic purposes, a lumbar puncture was performed. Cerebrospinal fluid (CSF) analysis excluded neuroinfection. Blood tests and CSF were examined according to the demyelinating disease protocol. Oligoclonal antibodies were negative in both the CSF and blood serum. Aquaporin-4 (AQP-4) antibodies were not found in the blood serum, and myelin oligodendrocyte glycoprotein (MOG) antibodies were also negative.

The patient was discharged a week later in satisfactory condition. It was recommended to perform an MRI of the cervical and thoracic spine with contrast to rule out demyelinating lesions in other locations. After the imaging scans, a consultation was planned on the multiple sclerosis board. The patient continued dexamethasone 0.5 mg tablets daily for 14 more days.

**Figure 6:**
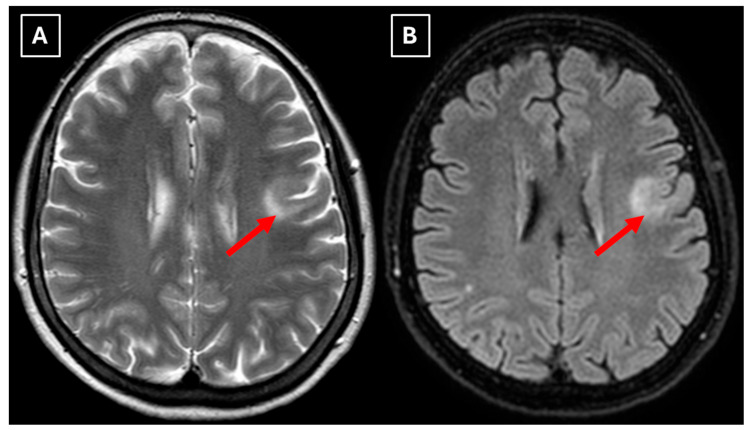
The 2-month follow-up MRI scan of the brain. In both (**A**): axial T2W axial sequence and (**B**): axial FLAIR, the mass has drastically decreased in size and has almost disappeared. A hyperintense demyelination focus can be seen subcortically in the basal parts of the left frontal lobe, which extends more into the white matter and has no mass effect on the surrounding structures. Red arrow—tumefactive demyelinating lesion.

**Figure 7:**
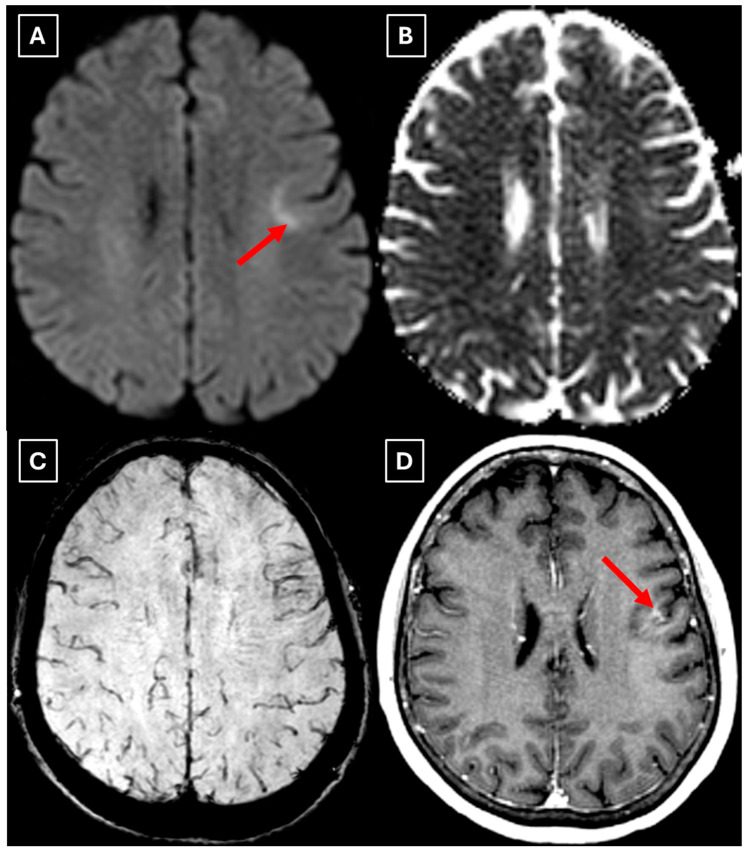
The 2-month follow-up MRI axial scan of the brain. Previously seen peripheral rim-like diffusion restriction is resolved and there is residual minimal vasogenic edema on the axial trace image (**A**). (**B**): ADC map. (**C**): SWI sequence shows no evidence of a left frontal lobe mass but a prominent central traversing vein in the lesion site. (**D**): T1 postcontrast sequence shows minimal patchy contrast enhancement along the margin of the lesion. Based on the observed changes over the past 2 months, it is most likely that the findings correspond to a demyelinating lesion. Red arrow—tumefactive demyelinating lesion.

Two months later a follow-up head MRI was performed. It showed a substantial decrease in the volume of the lesion, no mass effect on the surrounding structures, and no perifocal edema (Figure 6 and Figure 7). No other demyelinating lesions were found in the cervical and thoracic spine MRI. The multiple sclerosis board recommended an MRI follow-up after six months as well as a clinical evaluation of the patient.

## 3. Discussion

TDLs are typically related to MS with an estimated incidence of approximately 1–3 per 1000 MS cases. However, this is likely an underestimation due to the lack of a global MS registry [10]. Despite the strong relation with MS, TDLs can also occur as isolated, monophasic events or in other demyelinating diseases such as myelin oligodendrocyte glycoprotein antibody-associated disease (MOGAD), neuromyelitis optica spectrum disorder (NMOSD), and acute disseminated encephalomyelitis (ADEM), though this is considered rare. In addition, TDLs have been reported following viral infections such as HIV and the use of certain immunomodulatory drugs such as tacrolimus and natalizumab [3,10]. In this case, the lesion represented the patient’s first demyelinating episode without prior medical history or radiological evidence of MS, as well as other known demyelinating diseases, viral infections, or exposure to immunomodulatory agents.

Compared to previously reported cases, this case report highlights several distinguishing features. First, no other specific demyelinating lesions were identified to suggest a demyelinating disease, which is atypical since TDLs are in general co-occurring with other demyelinating foci [1,11]. Lucchinetti et al. (2008) [1] conducted the largest series of a biopsy-proven TDL study, and it was reported that 70% of patients with pathologically confirmed TDLs already had multiple lesions on pre-biopsy MRI, with a similar percentage developing definitive MS later. The detection of additional demyelinating lesions might act as an early radiological indicator of dissemination and a higher likelihood of progression into MS.

Secondly, the presence of a T2/FLAIR mismatch sign in this case, which initially was suggestive of a low-grade astrocytoma, adds diagnostic complexity and underlines the potential for radiological overlap between TDLs and IDH-mutant astrocytomas. One proposed pathophysiological explanation of this overlap is the presence of microcysts and enlarged intracellular space with fluid characteristics similar to cerebrospinal fluid [12,15]. Such components may also be present in both low-grade gliomas and TDLs, resulting in marked T2 hyperintensity and relative suppression on FLAIR sequences. Additionally, this apparent overlap with TDLs may be further explained by shared microstructural changes such as severe myelin loss, reactive gliosis, and accumulation of macrophages, as well commonly seen edema, similarly producing an imaging appearance of (IDH)-mutant 1p/19q non-coded astrocytoma [16]. Studies have also shown that low-grade astrocytomas are among the most frequent histopathological misdiagnoses of TDLs, reflecting their morphological similarity [1]. However, it remains uncertain whether analogous microstructural or fluid-dynamic mechanisms take place in TDLs, since there is no research available that directly investigated the histopathological correlates of the T2/FLAIR mismatch sign in TDLs; in addition, this sign is not mentioned in MRI features of demyelinating diseases. Therefore, its application, specificity, and sensitivity for TDL diagnosis are not established. The underlying mechanisms of TDL pathophysiology also remain incompletely understood, but immune-mediated inflammation with massive infiltration of macrophages and lymphocytes, dysregulated astrocyte signaling, complement activation arising as part of multiple sclerosis leading to exaggerated CNS immune responses, and medication effects such as fingolimod use, as well as its cessation, have all been proposed as contributing factors [6,10,11].

Finally, we provide a high-resolution MRI follow-up of two months’ duration showing near-complete regression of the lesion after corticosteroid therapy. The lesion decreased from 37.34 mm × 34.85 mm (AP × LL) to 24.87 mm × 29.71 mm following two weeks of corticosteroid therapy and after almost two months from the first MRI scan to minimal patchy contrast enhancement (Figure 7D). This marked decrease in the size of the lesion gives strong evidence that a demyelinating rather than neoplastic process is present.

To emphasize the distinctive aspects of this case, we created a comparative summary (Table 1) of previously reported cases of tumefactive demyelinating and neoplastic lesions presenting diagnostic overlap. A review of the literature disclosed that reported cases of TDLs presenting with a T2/FLAIR mismatch sign are exceptionally rare, with only one reported case to date by Le et al. (2023) [16]. To provide broader context, we included additional reported cases where larger than 2 cm focal lesions were first thought to be recognized as gliomas based on radiological and clinical presentation, as well as a contrasting case reported by Petrašová et al. (2024) [17] where a presumed TDL was subsequently identified as anaplastic astrocytoma on biopsy. To identify relevant cases, we conducted a PubMed search using the keywords “tumefactive demyelinating lesions,” “multiple sclerosis,” “astrocytoma”, and “CNS tumors”. This overview highlights key variations in terms of the presence of the T2/FLAIR mismatch sign, contrast enhancement, mass effect, additional demyelinating MS foci, response to steroids and biopsy, and laboratory findings such as oligoclonal bands and MOG-IgG seropositivity.

Although biopsy remains the diagnostic gold standard for TDLs, it may be infeasible or pose significant risks depending on the location of the lesion, as well as carry postoperative complications. MRI is the main diagnostic modality in the evaluation of tumefactive demyelination lesions, sparing patients from having a biopsy in certain cases. Magnetic resonance spectroscopy (MRS) provides metabolic information showing a high level of choline and a low level of N-acetylaspartate in TDLs. However, similar changes in metabolism are found in neoplastic lesions, making differentiation challenging as it is based only on elevated choline and reduced N-acetylaspartate findings.

There are no pathognomonic features for tumefactive demyelinating lesions, but there are still some MRI findings that support the diagnosis. TDLs appear larger than 2 cm and are most often located in the frontal or parietal regions. The supratentorial white matter, especially in subcortical and periventricular locations, is commonly affected. On T1-weighted images they usually demonstrate hypointensity and hyperintensity on T2-weighted images, consistent with demyelination. These lesions have a varying degree of perifocal edema and mass effect, but both are usually mild relative to the lesion size in comparison to neoplasms or abscesses. The most characteristic enhancement pattern is the open-ring (incomplete rim) enhancement, which is significantly more common in TDLs than closed-ring enhancement. The open ring typically faces the cortex or gray matter and is considered a strong supportive feature for TDL diagnosis [3,11,18]. Contrast enhancement is not a universal feature, as Lucchinetti et al. (2008) [1] reported that approximately 5% of TDLs on postcontrast T1-weighted MRI did not show enhancement in their study. This finding was also observed in this case (Figure 3D), as well as in the case presented by Zafar et al. (2022) [19]. Therefore, the absence of enhancement does not necessarily exclude a demyelinating pathology.

Another MRI feature strongly associated with TDLs is a central vein sign (CVS), which was also observed in this case (Figure 3, Figure 5, and Figure 7), currently being considered for inclusion in MS diagnostic criteria. On SWI or T2-weighted sequences, CVS appears as a thin hypointense line or dot less than 2 mm in diameter, visible in at least two planes, and running partially or completely through the center of a lesion [2]. Although most studies of the CVS have focused on MS and radiologically isolated syndrome, the CVS is also applicable for TDLs, representing an atypical variant of MS. In radiologically isolated syndrome, Suthiphosuwan et al. (2019) [21] reported a median of 87% CVS-positive lesions per case, underscoring its strong association with inflammatory demyelination [2]. In the TDL cohort described by Ongphichetmetha et al. (2024) [2], CVS was observed in approximately half of the patients, while open-ring enhancement occurred at a similar frequency of around 50%, with both features supporting an inflammatory demyelinating rather than neoplastic process.

We also found similarities between this case and the classical imaging features of TDLs described in the literature. Hypoattenuation on noncontrast CT corresponding to enhancing areas on MRI is another useful imaging finding in TDLs [10], which may be explained by the tissue rarefaction caused by the active demyelination rather than increased cellularity, which is seen in tumors, thereby supporting the use of MRI with noncontrast CT in TDL diagnosis. Furthermore, restricted diffusion along the lesion margin with elevated central ADC values, in contrast to neoplasms which more commonly show central diffusion restriction, have been described as a commonly presenting feature of TDLs [3,4,10], which was also observed in this case (Figure 5A,B).

Spinal MRI can be helpful for evaluating additional demyelination specific to multiple sclerosis, since spinal cord lesions associated with MS have been documented in TDLs. In a cohort of patients with TDLs, 43% had spinal cord lesions characteristic of MS on MRI [22].

Additionally, MOGAD should be considered in patients presenting with an isolated lesion without typical imaging features of MS. Cacciaguerra et al. (2023) [23] reported a higher frequency of TDLs among MOGAD patients, supporting the importance of evaluating such lesions for MOGAD to establish correct diagnosis, guide appropriate therapy, and provide prognostic information. MRI features of MOGAD-associated TDLs are characterized by poorly demarcated borders with a so-called “fluffy appearance”, frequently located in the posterior fossa and deep gray matter, in contrast to MS-related TDLs, which more commonly involve the hemispheric white matter. The absence of classic MS imaging features, such as a T2-hypointense rim, T1 hypointensity, DWI restriction, or ring contrast enhancement, also favors MOGAD. Although both can present with large lesions, mass effect, and edema, TDLs associated with MS are more likely to have additional demyelinating lesions or positive oligoclonal bands in cerebrospinal fluid. The study has further reported complete lesion resolution in more than half of patients with MOGAD-associated TDLs on MRI follow-up, whereas such resolution was less common in MS, making complete lesion resolution a reliable hallmark of MOGAD [23]. Serological testing and lumbar puncture for detecting oligoclonal bands and MOG antibodies can be informative in establishing the correct diagnosis before considering a biopsy.

MRI findings alone can be misleading, but in correlation with the response to corticosteroid therapy, they may point towards a demyelinating process. TDLs may show significant reduction or near-complete resolution on follow-up imaging after corticosteroid therapy, suggesting their non-neoplastic etiology. As illustrated by the cases summarized in Table 1, similar observations in the literature have also been documented. Turkistani et al. (2018) [18] described a lesion with open-ring enhancement and moderate mass effect which initially suggested a glioma but underwent a decrease in size following corticosteroid therapy. Zafar et al. (2022) [19] documented a non-enhancing lesion in the parietal lobe without mass effect or other additional lesions, initially suspected to represent a low-grade glioma but which otherwise showed considerable radiological regression following corticosteroid therapy, thus suggesting its demyelinating origin.

It is worth mentioning that some TDLs may only partially respond to corticosteroid therapy, and recurrence or even lesion progression can still occur despite therapy [24]. Corticosteroids have also been shown to transiently induce size and edema reduction in CNS neoplasms such as CNSL, with the possibility of recurrence following cessation of therapy, leading to a potential delay in reaching the correct diagnosis [18,25]. Because both TDLs and CNSLs can regress after corticosteroid therapy, the corticosteroid response alone should not be used as a distinguishing factor between demyelination and neoplasm. In addition, histopathological evaluation may have important limitations. Biopsy performed after corticosteroid therapy can yield misleading samples, as CNSL can exhibit inflammation and demyelination changes that are histopathologically similar to demyelinating diseases. Even in the absence of prior corticosteroid therapy, early lesions of CNSL can also show demyelination with sparse or absent tumor cells, leading to non-diagnostic biopsy samples and requiring a repeat biopsy for a definitive diagnosis [25,26]. It is suggested that short-interval MRI follow-up of these lesions should be considered due to possible recurrence or further progression to definitive MS [2,19].

Le et al. (2023) [16] presented a similar case where a TDL demonstrated the T2/FLAIR mismatch sign. The authors emphasized that when applied outside glioma-focused cohorts, the specificity of this sign substantially decreases, as for example, several reports have been described in non-neoplastic etiologies and pediatric tumors [13]. Additionally, the T2/FLAIR mismatch sign should not be interpreted in isolation, especially when cystic or necrotic components and contrast enhancement are present [16].

In this case, despite the presence of a T2/FLAIR mismatch sign (Figure 2), several imaging characteristics were more suggestive of a TDL rather than IDH-mutant astrocytoma. The lesion was located in juxtacortical white matter and had cortical sparing, which is unusual for IDH-mutant astrocytomas, since they typically have been shown to infiltrate and expand the cortex. The diffusion restriction at the lesion margin with centrally elevated ADC values was more consistent with reported DWI findings of TDLs and contrasted with the central restriction often seen in gliomas. The mass effect was mild in comparison to the overall lesion size, favoring demyelination rather than neoplasm in this case. Additionally, the lesion had a central vein sign, as described above, a radiological sign supportive of inflammatory demyelination. The combination of cortical sparing, peripheral diffusion restriction, mild mass effect, and the appearance of central vein sign represented strong features that raised suspicion of a tumefactive demyelination lesion.

Follow-up MRI showed lesion shrinkage after two weeks (Figure 4) and by two months, near-complete lesion regression and resolution of perifocal edema. The radiological course was typical for TDLs and no alternative differential diagnosis appeared reasonable, as both the clinical response to therapy and MRI follow-up strongly supported the demyelinating origin. Thus, biopsy was not performed in this case. In the literature similar cases have been reported where biopsy was avoided, as the disease responded to treatment and imaging findings were consistent with a demyelinating process [27]. Moreover, biopsy of the brain in TDLs carries considerable procedural risks, including seizures and post-operative infections [2]. However, despite the lesion regression after corticosteroid therapy, which was supportive of a TDL diagnosis, the lack of biopsy confirmation represents a limitation in this case and warrants explicit acknowledgment of the risk of misdiagnosis, especially with lesions that have less certain regression.

Currently, clear guidelines for the follow-up imaging of tumefactive demyelinating lesions are lacking and the available evidence remains scarce. We suggest short-interval MRI follow-up of TDLs within two to eight weeks after initial treatment to assess treatment response, adjust the treatment strategy if needed, and reevaluate the lesion. Additionally, long-term MRI surveillance should be considered due to the risk of lesion recurrence, progression into definitive MS, and the potential for misdiagnosis.

## 4. Conclusions

A tumefactive demyelinating lesion with a T2/FLAIR mismatch sign represents an exceptionally rare diagnostic pitfall. Even when this radiological sign is present, such lesions should be primarily evaluated in the context of adult-type gliomas and as a TDL only in carefully selected cases that show radiological features suggesting demyelination. Integration of corticosteroid response can help avoid biopsy, which carries considerable risks, but it must be interpreted with caution. Close clinical monitoring and short-interval MRI follow-up are essential for patients who do not undergo biopsy.

## Figures and Tables

**Table 1:** Comparative overview of cases of tumefactive demyelinating and neoplastic lesions presenting diagnostic overlap on MRI.

Author (Year)	Age/Sex	Initial Diagnosis	Laboratory Analysis	T2/FLAIR Mismatch Sign	T1W1 Postcontrast Enhancement	Mass Effect	Additional MS Foci	Response to Steroids	Biopsy	Diagnosis
This case (2025)	63/F	Low-grade astrocytoma	Negative	Yes	No	None	No	Yes	No	TDL
Le et al. [16] (2023)	46/M	Low-grade astrocytoma	Positive OCBs	Yes	Yes	Present	Yes	Yes	No	TDL
Turkistani et al. [18] (2018)	37/F	Glioma	Positive OCBs	No	Yes	Present	Yes	Yes	No	TDL
Zafar et al. [19] (2022)	40/F	Glioma	Not known	No	No	None	No	Yes	No	TDL
Slaghour et al. [20] (2020)	29/F	Low-grade astrocytoma	Not known	Yes	No	None	Not known	Not known	Yes	IDH-mutant diffuse astrocytoma (grade II)
Petrašová et al. [17] (2024)	29/F	TDL	Positive OCBs	No	No	Present	Yes	No	Yes	Anaplastic astrocytoma (grade III)

M—male, F—female, MS—multiple sclerosis, TDL—tumefactive demyelinating lesion, OCBs—oligoclonal bands, IDH–isocitrate dehydrogenase.

## Data Availability

The data presented in this study are available on request from the corresponding author due to privacy restrictions.

## Abbreviations

The following abbreviations are used in this manuscript:

TDL	Tumefactive demyelinating lesion
MS	Multiple sclerosis
CNSL	Central nervous system lymphoma
CNS	Central nervous system
FLAIR	Fluid-attenuated inversion recovery
CT	Computed tomography
DWI	Diffusion-weighted imaging
IDH	Isocitrate dehydrogenase
MOGAD	Myelin oligodendrocyte glycoprotein antibody-associated disease
NMOSD	Neuromyelitis optica spectrum disorder
ADEM	Acute disseminated encephalomyelitis
HIV	Human immunodeficiency virus
MRS	Magnetic resonance spectroscopy
CVS	Central vein sign
